# Management of Postoperative Pain after Elective Craniotomy: A Prospective Randomized Controlled Trial of a Neurosurgical Enhanced Recovery after Surgery (ERAS) Program

**DOI:** 10.7150/ijms.46403

**Published:** 2020-06-21

**Authors:** Liang Qu, Bolin Liu, Haitao Zhang, Eric W. Sankey, Wei Chai, Binrong Wang, Zhengmin Li, Jiangtao Niu, Binfang Zhao, Xue Jiang, Lin Ye, Lanfu Zhao, Yufu Zhang, Tao Zheng, Yafei Xue, Lei Chen, Long Chen, Haijing Han, Wenjuan Liu, Ruigang Li, Guodong Gao, Xuelian Wang, Yuan Wang, Shiming He

**Affiliations:** 1 Department of Neurosurgery, Tangdu Hospital, Fourth Military Medical University. Xi'an, China.; 2 Department of Neurosurgery, Xi'an International Medical Center, Xi'an, Shaanxi, China.; 3 Department of Respiratory and Critical Care Medicine, Tangdu Hospital, Fourth Military Medical University. Xi'an, China.; 4 Department of Anesthesiology, Xi'an International Medical Center, Xi'an, Shaanxi, China.; 5 Department of Anesthesiology, Tangdu Hospital, Fourth Military Medical University. Xi'an, China.; 6 Department of Nutrition, Tangdu Hospital, Fourth Military Medical University. Xi'an, China.; 7 Duke University Hospital. Durham, NC. USA.

**Keywords:** Enhanced recovery after surgery (ERAS), neurosurgery, elective craniotomy, pain management, outcomes

## Abstract

**Objective:** To prospectively evaluate the efficacy of a neurosurgical enhanced recovery after surgery (ERAS) protocol on the management of postoperative pain after elective craniotomies.

**Methods:** This randomized controlled trial was conducted in the neurosurgical center of Tangdu Hospital (Fourth Military Medical University, Xi'an, China). A total of 129 patients undergoing craniotomies between October 2016 and July 2017 were enrolled in a randomized clinical trial comparing an ERAS protocol to a conventional postoperative care regimen. The primary outcome was the postoperative pain score assessed by a verbal numerical rating scale (NRS).

**Results:** Patients in the ERAS group had a significant reduction in their postoperative pain scores on POD 1 compared to patients in the control group (p < 0.05). More patients (n = 44, 68.8%) in the ERAS group experienced mild pain (NRS: 1 to 3) on POD1 compared with patients (n = 23, 35.4%) in the control group (p < 0.05). A further reduction in pain scores was also observed on POD 2 and maintained on POD 3 in the ERAS group compared with that in the control group. In addition, the median postoperative length of hospital stay was significantly decreased with the incorporation of the ERAS protocol compared to controls (ERAS: 4 days, control: 7 days, P<0.001).

**Conclusion:** The implementation of a neurosurgical ERAS protocol for elective craniotomy patients has significant benefits in alleviating postoperative pain and enhancing recovery leading to early discharge after surgery compared to conventional care. Further evaluation of this protocol in larger, multi-center studies is warranted.

## Introduction

Enhanced recovery after surgery (ERAS) protocol are evidence-based perioperative guides that promote stress reduction and make substantial efforts to optimize patient outcomes by systematically addressing modifiable pre-, peri-, and post-operative factors 1, 2. In recent decades, a number of standardized enhanced recovery-after-surgery (ERAS) protocols have been implemented at every stage of the perioperative process 3-7. The concept of ERAS was established to standardize clinical practice, improve functional capacity after surgery, speed up the patients' rehabilitation, reduce postoperative length of stay (LOS), reduce medical cost, and improve the patients' satisfaction 3, 8, 9.

When making the decision on whether to adopt a new comprehensive protocol in elective craniotomies, neurosurgeon must consider the quality & safety of the procedure and risk tolerance 2, 10, 11. Moreover, the quality improvement methods should raise the degree of patients' perceived comfort 12, 13. Acute pain is common during the postoperative period, and is associated with complications and adverse outcomes 14, 15. To date, there is controversy in the literature regarding the evaluation of pain and its intensity in patients undergoing neurosurgical procedures 16, 17. Moreover, different types of pain therapy have been advocated for the same neurosurgical procedure based on clinicians' personal or institutional preference 17-20. To our knowledge, no well-designed study has been conducted to compare the effect on perioperative verbal numerical rating scale (NRS) scores under a focused ERAS program versus conventional management.

The utilization of ERAS protocols in neurosurgery remains limited 1. Although there are a few studies to evaluate new protocols for elective spinal and peripheral nerve surgery, the quality and safety outcomes of those programs have not been well described 21, 22. Recently, our group has developed a multi-disciplinary neurosurgical ERAS protocol for elective craniotomies based on the best available evidence 1, 21, 22. Our perioperative care team included neurosurgeons, anesthetists, residents, operating room nurses, neurophysiologists, dieticians and other non-medical staff 1. This ERAS approach links patients, clinicians and scientists in a new way that aims to make improvements in healthcare cost, quality and timeliness. By implementing an evidence-based neurosurgical ERAS protocol among 129 patients undergoing craniotomies at the Neurosurgical center of Tangdu Hospital, Fourth Military Medical University (Xi'an, China), we evaluated the impact of this protocol on postoperative pain control by analyzing data on pain intensity and pain characteristics.

## Methods

This study was approved by the Ethical Committee of Tangdu Hospital at the Fourth Military Medical University, and this study has been registered in the Chinese Clinical Trial Registry with registration number ChiCTR-INR-16009662.

### The intervention: ERAS protocol and conventional protocol

All patients were randomized 1:1 to receive their perioperative care under our novel ERAS protocol versus conventional care. Details of our neurosurgical ERAS protocol for patients undergoing elective craniotomy was reported in our previous study^1^. Care providers for patients in the ERAS group were instructed to record various clinical outcomes data and to implement as many items of the ERAS protocol as much as possible (**Supplementary file 1** and** Supplementary file 2**). Care was implemented according to individual discretions of the neurosurgeons and anesthetists, based on routine institutional neurosurgical postoperative protocols for all patients in the conventional protocol group (**Supplementary file 3**). Patients were followed until at least 4 months after hospital discharge or until the time of death.

### Compliance with ethical standards

Informed consent was achieved from all individual participants included in this study or their legal representatives. The analysis and usage of patient information for this study was approved by the Ethical Committee of Tangdu Hospital. And the methods were carried out in accordance with the approved guidelines. This randomized control trial (RCT) was registered at Chinese Clinical Trial Registry (Registration date: October 27, 2016, http://www.chictr.org.cn/showproj.aspx?proj=16480).

### Study participants

The inclusion criteria were as follows: (1) Patients with a single intracranial lesion and medical eligibility for elective craniotomy; (2) Age between 18-65 years; (3) Patients who are able to communicate well with the medical staff; (4) Patients who understood and signed an Informed Consent, with good compliance in the study.

The exclusion criteria comprised of (1) non-brain tumor patients, such as severe craniocerebral injury leading to bilateral mydriasis, unstable vital signs; (2) children (patients less than 18 years); (3) awake craniotomy; (3) patients with severe spinal cord shock; (4) other trauma caused by preoperative cardiac arrest, combined with severe limb fractures or thoracic and abdominal injury; (5) infection or inflammation in the surgical area; (6) serious comorbidities (blood system, respiratory system, digestive system, etc.) patients; (7) patients with severe heart disease (such as coronary heart disease, myocardial infarction, etc.); (8) patients with ULN and/or renal function (Cr)> 1.5 times ULN with liver function (ALT, AST)> 2 times; (9) patients with mental illness; (10) women who have a childcare plan within 6 months of pregnancy or breastfeeding; (11) other patients who were considered unsuitable for inclusion in the study.

### Patient enrolment

Research assistants (RAs) consulted duty nurses daily to identify all new admissions as potential study participants. After confirming eligibility and obtaining consent, RAs collected patient characteristics data including demographic information (age, sex), admission diagnosis, preoperative co-morbidity status (American association of anesthesiologists grades, ASA grades) and other presenting physical characteristics (smoking, diabetes, motion sickness, hypertensive disease, etc.). Data about the details of operations like types of operation, lesion locations (supratentorial superficial lesion, supratentorial deep-seated lesion or infratentorial lesion) were also assessed. All data were collected on a secure, web-based program.

### Statistical analysis

Data were collected during the hospitalization and at the 4-month follow-up after hospital discharge. Descriptive statistics of the ERAS and control groups were compared for all relevant patient characteristics. A sample size of at least 60 patients per arm was calculated to have a power of 80% and a significance of 5%. To compensate for potential dropouts, 129 patients were enrolled. Continuous data with a normal distribution were statistically tested for group differences using *chi*-square test and Fisher's exact text. The statistical analysis was performed with SPSS software (Ver. 19, IBM Corp., Armonk, NY). A *P* value of <0.05 was considered to be statistically significant.

### Randomization

After obtaining informed consents, patients were prospectively randomized into two groups (1:1 ration) by simple randomization procedures (computerized random numbers) by the research coordinator. A total of 65 patients were allocated to control group who received the conventional perioperative care, whereas 64 patients were allocated to ERAS group who received care according to the neurosurgical ERAS protocol. Due to the requirement for active patient participation, it was not possible to perform the study with blinded participants and care providers. Only those who collected and assessed outcomes were blinded to the allocation.

### Outcome measurements

The primary outcome of this study was the patients' postoperative pain NRS scores. The verbal NRS ranges from 0 to 10 (0 represents no pain and 10 represents the worst pain). Postoperative NRS of surgical site pain was first assessed on postoperative day (POD) 1 and repeated daily until the patient had no complaint of pain or was discharged.

Secondary outcome measures included: (1) analgesic, nonanalgesic medication administration, weak opioid analgesics ( + nonopioid analgesic drugs) and strong opioid analgesics ( + nonopioid analgesic drugs) which were administered for postoperative pain treatment depending on the assessment and decision of the care team; (2) total hospital length of stay from admission to discharge; (3) post-procedure length of stay from end of procedure to discharge, and (4) total cost of hospitalization (CNY).

## Results

### Baseline characteristics

**Figure 1:**
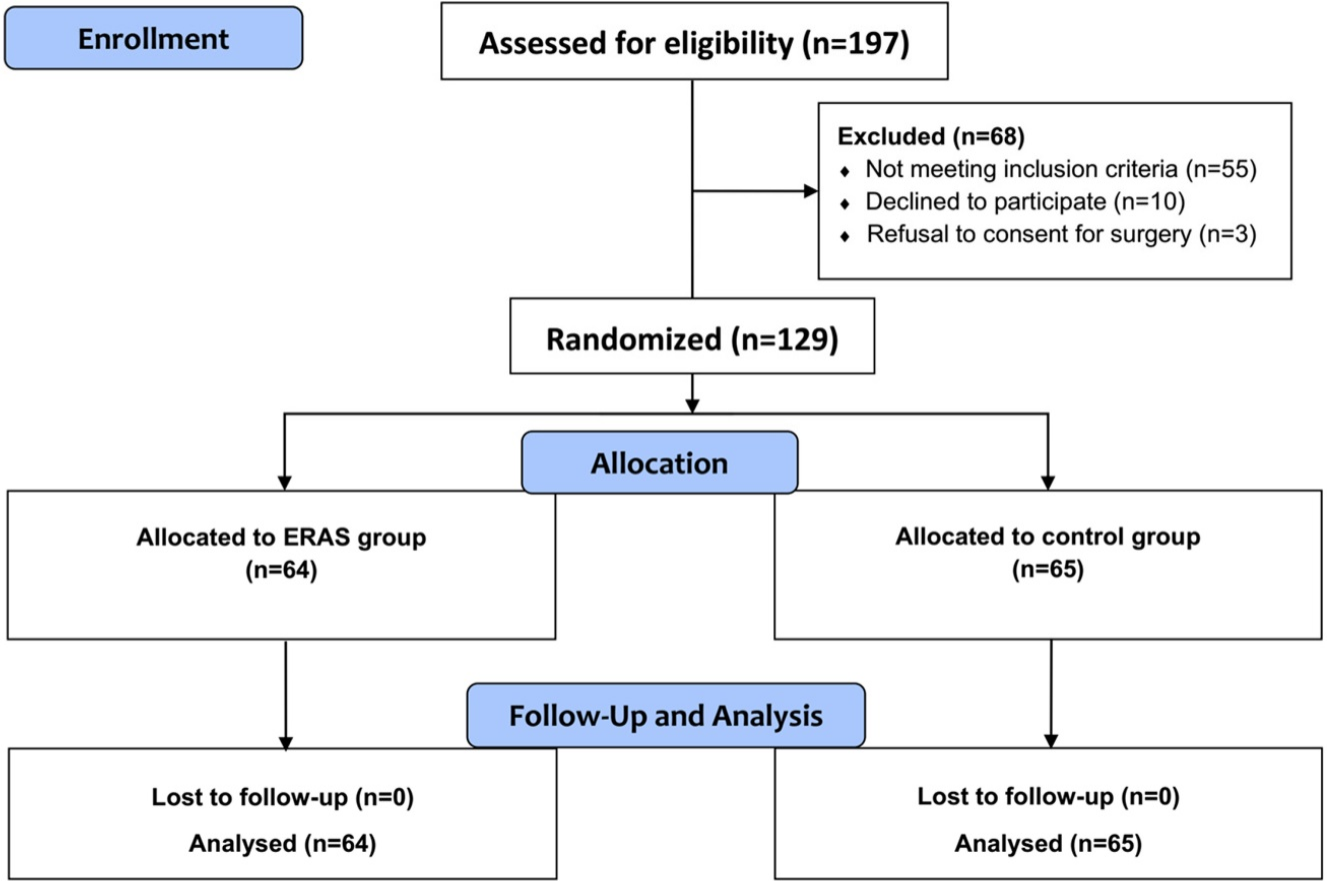
** Flow diagram of CONSORT study design.** Randomized controlled trial comparing ERAS group versus control group for elective craniotomies.

From October 2016 to July 2017, 129 patients aged 18 to 65 years, who were admitted for elective craniotomies at Department of Neurosurgery, Tangdu Hospital were enrolled for this study. A total of 129 patients (64 in the ERAS group and 65 in the control group) were enrolled in this study and were preoperatively randomized to one of two groups: the ERAS or the control group. Patient characteristics are shown in **Table 1**. Demographic and clinical features were not significantly different between the intervention and control groups. All patients in both groups underwent elective craniotomies by the same experienced neurosurgical team (**Figure 1**). In both groups, the proportion of female patients was higher than that of male patients, but there was no significant gender difference between two groups. The relevant details of surgery and outcomes are also shown in **Table 1**. There was no significant difference in categories of indications for operations. Patients who met the inclusion criteria were included in the study and presented with common neurological deficits in both groups. Lesion location was not significantly different between the groups, and every patient went through the standardized surgical procedure regimen as mentioned previously.

### The assessment of postoperative surgical pain

Primary outcome measurements are shown in **Table 2**. Patients in the ERAS group had a significant reduction in postoperative pain scores on POD 1 compared to patients in the control group (p < 0.05). In addition, more patients (n = 44, 68.8%) in the ERAS group experienced mild pain (NRS: 1 to 3) on POD1 compared with that (n = 23, 35.4%) in the control group. Similarly, less patients (n = 18, 28.1%) in the ERAS group experienced moderate pain (NRS: 4 to 7) on POD1 compared with that (n = 39, 60.0%) in the control group. A total of 3.1% (n = 2) of patients experienced severe pain (NRS: 8 to 10) on POD1 in the ERAS group, while 4.6% (n = 3) of patients experienced severe pain (NRS: 8 to 10) on POD1 in the control group. A significant reduction in pain score was observed on POD 2 and POD 3 in the ERAS group compared with that in the control group (p < 0.05). The duration of time that patient's complained about postoperative pain was also shortened for the patients in the ERAS group compared with the control group (p < 0.001, **Table 2**). The majority of patients reported resolution of postoperative pain after only 1-2 days in the ERAS group, which remained present in the control group (54.7% in ERAS vs. 20.0% in control, p < 0.05).Similarly, less patients had a significant complaint of continued pain after 2-3 days in the ERAS group compared to the control group (21.9% in ERAS vs. 40.0% in control, p < 0.05).

### Analgesic medication administration and other secondary outcomes

Analgesics were administered to relieve postoperative pain depending on the assessment and decision of the attending team. The analgesics were divided into three categories (WHO classification of pain treatment,** Table 3**). The analgesic medications used in the ERAS group and control group are shown in **Table 4**. In general, the number of patients receiving WHO Class I - WHO Class III medication was not significantly different between two groups (ERAS group: n=15, 23.4% vs. control group: n=22, 33.8%, P =0.356). On POD 1, the percentage of patients receiving WHO Class I analgesic medications was 14.1% in the ERAS group vs. 12.3% in the control group. The percentage of patients receiving WHO Class II analgesic medication was 4.7% in the ERAS group vs. 13.8% in the control group. Lastly, the number of patients receiving WHO Class III analgesic medication was 4.7% in the ERAS group vs. 7.7% in the control group. This suggested that patients in the control group required stronger analgesia than patients in the ERAS group, similar to the results shown by their NRS scores.

Other secondary outcome measurements are shown in **Table 4**. The median of total hospital LOS was significantly reduced from 13 days in the control group to 10 days in the ERAS group (P = 0.004). The median of postoperative LOS was also significantly reduced from 7 days in the control group to 4 days in the ERAS group (P < 0.001). In addition, the total cost of hospitalization was RMB 52424 (range: 33652-118965) in the ERAS group and RMB 64462 (range: 39973-141216) in the control group (P < 0.001).

### Postoperative Complications and Re-admission

Postoperative complications are listed in **Table 5**. 9 patients (14.1%) in the ERAS group and 14 patients (21.5%) in the control group had postoperative fever of up to 38 °C (p=0.358). However, their temperature returned to normal within 48 hours postoperatively after removal of their urinary and central venous catheters. Three patients in the ERAS group and 2 patients in the control group had a postoperative seizure (p=0.680). None of the patients experienced symptoms of significantly raised intracranial pressure, required surgical revision, suffered mental status changes or need for emergent imaging, diabetes insipidus and toxic epidermal necrolysis (due to phenytoin) in the ERAS group. Four patients (6.3%) in the ERAS group and 3 patients (4.6%) in the control group were noted to have blood sugar levels of >200 mg/dL intraoperatively (p = 0.718), and this trend persisted for 3 days postoperatively due to the use of high dose steroid-therapy in all patients, warranting the use of short-acting insulin therapy. Ten patients (15.6%) in the ERAS group and 18 patients (27.7%) in the control group had nausea (moderate to severe) (p = 0.135). Ten patients (15.6%) in the ERAS group and 20 patients (30.8%) in the control group had anti-emetics treatment (p=0.060). And none of the patients in the two groups required re-admission during the 4-month follow-up period.

## Discussion

In order to assess the impact of our novel, neurosurgical ERAS protocol for elective craniotomies on postoperative pain, we analyzed data on pain intensity and pain character among 129 patients randomized to perioperative management via the ERAS protocol vs. conventional care. The results of this study suggest that multidisciplinary cooperation under a structured ERAS protocol may help alleviate postoperative pain, reduce total hospital LOS and postoperative LOS, and reduce the total cost of medical care.

Craniotomy is a relatively common surgical procedure with a high incidence of postoperative pain 10, 23. Development of standardized pain management and ERAS protocols may help optimize patient-reported outcomes and reduce health care costs 1, 23. The majority of reported ERAS programs for pain management dependent multidisciplinary cooperation, which include the efforts of neurosurgeons, anesthetists, residents, operating room nurses, neurophysiologist, dieticians and the support from family members of the patient 1, 24-26. However, these studies vary widely in their methodology and targeted patient populations. Some studies were limited by the generalization of implementing their recommendations in other medical institutions 25-28. Recently we have implemented a novel multidisciplinary, evidence-based, neurosurgical ERAS program for elective craniotomy patients in a single center 1. And evidences have suggested that the ERAS protocol could reduce the length of hospital stay postoperatively and that in turn enhanced the patients' recovery 29, 30. In this study, we still based on the same protocol, but with focusing on the postoperative pain control, which are vital component to the whole picture of current neurosurgical ERAS protocol.

Optimization of pain management is a key element of ERAS protocol 31. Till now, there is no consensus regarding the pain management and analgesic regimen for post-craniotomy pain 32. NRS is one of the most frequently used standardized methods to evaluate postoperative pain 31. In our study, in spite of the treatment of postoperative pain with analgesics, over 64.6% of the patients suffered from moderate-severe pain in the control group (**Table 2**). This is consistent with some previous reports on the prevalence of postcraniotomy pain 33-36, despite conventional treatment efforts. Our data showed that patients in the ERAS group had a statistically significant reduction in pain score on POD 1- POD 3 compared to patients in the control group (**Table 2**). Moreover, the incidence of moderate pain on POD 1 reduced with the implementation of the ERAS protocol, and the patients had shorter duration of pain complaints than those in the control group.

There is an intense debate on whether the ERAS program reduces pain after elective craniotomies 10. The main finding of this study was a trend for less pain in the ERAS group patients (**Table 2**). We speculate that the findings of reduced pain in this study may be related to some interventions included in the ERAS protocol such as smoking cessation, incisional local anesthetic blocks and additional use of acetaminophen/NSAIDs. In addition, multidisciplinary collaboration also reduces patient discomfort, speeds up wound healing, and thus reduces the degree and duration of postoperative pain 37. Firstly, one of the main interventions in ERAS protocol is smoking cessation 1. Smoking has been known to be harmful to overall health, and cigarette smoking may also be associated with a worse surgical outcome and prognosis in patients undergoing craniotomy 13. Some studies indicate that smoking cessation may reduce postoperative complications following craniotomy 38. Secondly, numerous studies have shown that scalp infiltration in patients undergoing craniotomies play crucial roles in post-craniotomy pain management 39-43. Accordingly, scalp infiltration with ropivacaine or bupivacaine in the ERAS protocol may reduce the incidence and severity of postoperative pain, which has also been shown in other studies 32, 44, 45. The mechanisms underlying the beneficial effects of local anesthetic blocks include a reduction in the inflammatory and stress response associated with surgery, lower levels of angiogenesis, a decrease in the requirements of volatile anesthetics and minimization or avoidance of opioid 46. Thirdly, non-opioid analgesics including acetaminophen or non-steroidal anti-inflammatory drugs were administrated according to the pain degree of patient postoperative NRS scores. Evidence has shown that opiates such as morphine are less effective for pain relief in craniotomy patients 47, 48. Therefore, postoperative morphine and equivalent opioids were not routinely prescribed due to their limited effect and wide ranges of side-effects for mild or moderate pain 12. The low dose consuming non-opioid analgesics can also reduce opioid consumption by 35-50%, and alleviate persistent pain without significant adverse effects 49-53. In our study, most patients experienced mild pain (NRS 1-3) on POD 1, and more patients showed shortened pain duration time (1-2d) in the ERAS group (**Table 2**). There was no statistical difference in analgesic medication administration between the two groups (p = 0.356, **Table 4**). These results supported the effectiveness of pain management protocol in the ERAS group, which had also improved the medical recovery of patients.

Hospital stay relies on various factors, which may be modified to a certain extent by the effect of perioperative care 54. Total hospital LOS and postoperative LOS was evaluated between the ERAS group and the control group (**Table 4**). The effectiveness of the ERAS protocol was confirmed with significant shorter hospital LOS and postoperative LOS in the ERAS group. Nonetheless, postoperative LOS and total LOS are affected by several demand factors (age, sex, disease severity, complications, *et al.*) and supply factors (clinical methodology, local medical insurance policies, bed occupancy, and *et al.*) 55, 56. These factors needed to be considered in assessing the efficacy of an ERAS protocol in clinical trials. Future studies may incorporate interventions designed to improve the comfort and use of individualized pain management for targeted patient populations. Future multicenter clinical trials for evaluating an evidence based neurosurgical ERAS protocol also require more rigorous design and power analysis, proper calibration for multiple comparisons, and the use of better outcome measures.

In addition, the current ERAS protocol incorporates nutritional interventions including preoperative carbohydrate loading and early restoration of oral solid food postoperatively, which may have a profound impact on the enhanced recovery 1, 57. Such interventions were shown to alleviate muscle loss and improve organ function such as pulmonary function in addition to improve glucose homeostasis and insulin resistance 58, 59. These beneficial effects may also correlate with a reduction in both hospital LOS and postoperative LOS in patients participating in an ERAS program for major surgeries including craniotomies 59, 60. We monitored all patients for postoperative complications and re-admission rates, and none of the patients had suffered from raised intracranial pressure, re-craniotomy, mental status changes (needs for emergent imaging), diabetes insipidus and toxic epidermal necrolysis (due to phenytoin) in the ERAS group (**Table 5**). However, 4 patients in the ERAS group had serial blood sugar levels >200 mg/dL intraoperatively which lasted for 3 days postoperatively and required insulin therapy. Limited by our case number, the current results may not reflect the influences of the ERAS protocol in this respect. To summarize, the postoperative complications and re-admission rate in the ERAS group was not increased as compared to that in the control group, while postoperative pain of patients was reduced significantly.

There are several limitations of the current study. First, subgroup analysis may be needed to perform with all consecutive patients within the ERAS group and conventional care protocol. Postoperative pain management is embedded in a multidisciplinary cooperation and the impact of pain management on recovery, pain relief, and length of stay needs to be interpreted in this context. Second, though postoperative pain was significantly reduced in the ERAS group, the use of opioid analgesics was not significantly decreased in the ERAS group compared to the control group. It is possible that expectations on the part of both the patients and researchers may cause bias towards a more favorable NRS score in the ERAS group since this study was not blinded. This limitation in interpretating the results of this study should be noted. Third, little information was assessed in-depth regarding the specific characteristics of targeted patient populations, which may be investigated in further studies. As mentioned in the Methods section, the ERAS pathway has been continuously adapted and updated during the study period to avoid the bias of various perioperative care pathways and unbalanced interventions.

## Conclusion

In conclusion, we have assessed the effect of an ERAS protocol for elective craniotomies, which includes a series of interventions, on alleviating postoperative pain and enhancing recovery after surgery. The results of this study suggest that our ERAS protocol may help to improve pain management after elective craniotomies when compared to conventional care measures. Moreover, the ERAS protocol also reduced total / postoperative hospital LOS and the total cost of medical care. There is an urgent need for larger multi-center studies to further evaluate this protocol in this unique patient population.

## Supplementary Material

Supplementary figures and tables.

## Figures and Tables

**Table 1:** Patient characteristics and details of operations

	Parameters	ERAS Group (n = 64)		Control Group (n = 65)		P
**Age (years)**						0.723
<40	patients, n (%)	38	59.4%	36	55.4%	
40-65	patients, n (%)	26	40.6%	29	44.6%	
**Gender**						0.713
Male	patients, n (%)	21	32.8%	24	36.9%	
Female	patients, n (%)	43	67.2%	41	63.1%	
BMI (kg/m^2^)	Median BMI, kg/m^2^ (Range)	24.1	15.9-29.6	24.7	19.1-28.4	0.395
**ASA classification**						0.358
ASA I	n (%)	9	14.1%	14	21.5%	
ASA II	n (%)	55	85.9%	51	78.5%	
**Concomitant diseases**						
Cardiac/hypertension	n (%)	13	18.8%	12	15.4%	0.827
Smoker	n (%)	6	10.9%	8	15.4%	0.778
Liver/gall bladder	n (%)	7	10.9%	4	7.7%	0.364
Lung	n (%)	5	7.8%	7	10.8%	0.763
Diabetes mellitus	n (%)	11	6.3%	7	7.7%	0.321
Miscellaneous	n (%)	13	4.7%	6	6.2%	0.087
Indication for surgery	Meningioma, n (%)	38	59.4%	30	46.2%	0.516
	Vestibular schwannoma, n (%)	7	10.9%	9	13.8%	
	CPA Cholesteatoma, n (%)	6	9.4%	8	12.3%	
	Glioma, n (%)	13	20.3%	18	27.7%	
Lesion location						0.566
	Supratentorial superficial	19	29.7%	16	24.6%	
	Supratentorial deep	23	35.9%	20	30.8%	
	Infratentorial	22	34.4%	28	43.1%	

ASA: American Society of Anesthesiologists. CPA: cerebellopontine angle.

**Table 2:** Primary outcome measures

	Parameters	ERAS Group (n = 64)		Control Group (n = 65)		P
Postoperative surgical pain, Mean (min-max)	Day of surgery	4.42	( 1-8 )	4.71	( 1-9 )	0.477
	POD 1	3.12	( 1-8 )	4.44	( 1-9 )	0.010
	POD 2	2.85	( 0-6 )	4.32	( 0-8 )	0.002
	POD 3	2.32	( 0-5 )	4.03	( 0-6 )	<0.001
	POD 4	2.25	( 0-4 )	2.83	( 0-6 )	0.273
POD1 Pain verbal NRS, n (%)						<0.001
	1-3	44	68.8%	23	35.4%	<0.001
	4-7	18	28.1%	39	60.0%	<0.001
	8-10	2	3.1%	3	4.6%	>0.999
Postoperative pain duration time, n (%)						<0.001
	1-2d	35	54.7%	13	20.0%	<0.001
	2-3d	14	21.9%	26	40.0%	0.026
	3-4d	13	20.3%	23	35.4%	0.056
	>4d	2	3.1%	3	4.6%	>0.999

**Table 3:** WHO classification of pain treatment

Class	Description	Examples
I	nonopioid analgesic drugs	nonsteroidal anti-inflammatory drugs, acetaminophen
II	weak opioids (+ nonopioid analgesic drugs)	tramadol, codeine
III	strong opioids (+ nonopioid analgesic drugs)	morphine, piritramid, meperidine

**Table 4:** Secondary Outcome measures

Parameter		ERAS Group (n = 64)		Control Group (n = 65)		*P*
Analgesic medication administration	Total pain treatment	15	23.4%	22	33.8%	0.356
	I	9	14.1%	8	12.3%	
	II	3	4.7%	9	13.8%	
	III	3	4.7%	5	7.7%	
Median total hospital lengthof stay from admission todischarge (days, min, 1st Q, 3rd Q, max)		10	4, 8, 12, 29	13	5, 11, 17, 34	0.004
Median post procedure lengthof stay from end of procedureto discharge (days, min, 1st Q, 3rd Q, max)		4	1, 3, 7, 13	7	3, 5, 11, 28	< 0.001
Total cost of hospitalization (CNY, min, 1st Q, 3rd Q, max)		52424	36652, 46210, 68863, 118965	64462	45973, 59641, 82623, 139153	< 0.001

**Table 5:** Postoperative Complications and Re-admission

Parameter	ERAS Group (n = 64)		Control Group (n = 65)		*P*
Postoperative fever	9	14.1%	14	21.5%	0.358
Postoperative seizure	3	4.7%	2	3.1%	0.680
Raised intracranial pressure	0		1		
Recraniotomy	0		0		
Mental status changes (needs for emergent imaging)	0		1		
Diabetes insipidus	0		0		
Toxic epidermal necrolysis (due to phenytoin)	0		0		
Postoperative blood sugar > 200 mg/dL	4	6.3%	3	4.6%	0.718
Nausea (moderate to severe)	10	15.6%	18	27.7%	0.135
Use of anti-emetics	10	15.6%	20	30.8%	0.060
Re-admission within 2 weeks after discharge	0		0		
Re-admission within 4 months after discharge	0		0		

## References

[1] Wang Y, Liu B, Zhao T, Zhao B, Yu D, Jiang X (2018). Safety and efficacy of a novel neurosurgical enhanced recovery after surgery protocol for elective craniotomy: a prospective randomized controlled trial. Journal of neurosurgery.

[2] Nanji KC, Ferris TG, Torchiana DF, Meyer GS (2013). Overarching goals: a strategy for improving healthcare quality and safety?. BMJ quality & safety.

[3] Cerantola Y, Valerio M, Persson B, Jichlinski P, Ljungqvist O, Hubner M (2013). Guidelines for perioperative care after radical cystectomy for bladder cancer: Enhanced Recovery After Surgery (ERAS((R))) society recommendations. Clinical nutrition.

[4] Melloul E, Hubner M, Scott M, Snowden C, Prentis J, Dejong CH (2016). Guidelines for Perioperative Care for Liver Surgery: Enhanced Recovery After Surgery (ERAS) Society Recommendations. World J Surg.

[5] Scott MJ, Baldini G, Fearon KC, Feldheiser A, Feldman LS, Gan TJ (2015). Enhanced Recovery After Surgery (ERAS) for gastrointestinal surgery, part 1: pathophysiological considerations. Acta anaesthesiologica Scandinavica.

[6] Mortensen K, Nilsson M, Slim K, Schafer M, Mariette C, Braga M (2014). Consensus guidelines for enhanced recovery after gastrectomy: Enhanced Recovery After Surgery (ERAS(R)) Society recommendations. The British journal of surgery.

[7] Thorell A, MacCormick AD, Awad S, Reynolds N, Roulin D, Demartines N (2016). Guidelines for Perioperative Care in Bariatric Surgery: Enhanced Recovery After Surgery (ERAS) Society Recommendations. World J Surg.

[8] Kehlet H, Wilmore DW (2002). Multimodal strategies to improve surgical outcome. American journal of surgery.

[9] Ljungqvist O, Scott M, Fearon KC (2017). Enhanced Recovery After Surgery: A Review. JAMA surgery.

[10] Tsaousi GG, Logan SW, Bilotta F (2017). Postoperative Pain Control Following Craniotomy: A Systematic Review of Recent Clinical Literature. Pain practice: the official journal of World Institute of Pain.

[11] Zhu M, Chang W, Jing L, Fan Y, Liang P, Zhang X (2019). Dual-modality optical diagnosis for precise in vivo identification of tumors in neurosurgery. Theranostics.

[12] Galvin IM, Levy R, Day AG, Gilron I (2019). Pharmacological interventions for the prevention of acute postoperative pain in adults following brain surgery. The Cochrane database of systematic reviews.

[13] Dunn LK, Naik BI, Nemergut EC, Durieux ME (2016). Post-Craniotomy Pain Management: Beyond Opioids. Current neurology and neuroscience reports.

[14] Ban VS, Bhoja R, McDonagh DL (2019). Multimodal analgesia for craniotomy. Current opinion in anaesthesiology.

[15] Li C, Sun W, Gu C, Yang Z, Quan N, Yang J (2018). Targeting ALDH2 for Therapeutic Interventions in Chronic Pain-Related Myocardial Ischemic Susceptibility. Theranostics.

[16] Dunbar PJ, Visco E, Lam AM (1999). Craniotomy procedures are associated with less analgesic requirements than other surgical procedures. Anesthesia and analgesia.

[17] Klimek M, Ubben JF, Ammann J, Borner U, Klein J, Verbrugge SJ (2006). Pain in neurosurgically treated patients: a prospective observational study. Journal of neurosurgery.

[18] Jeffrey HM, Charlton P, Mellor DJ, Moss E, Vucevic M (1999). Analgesia after intracranial surgery: a double-blind, prospective comparison of codeine and tramadol. British journal of anaesthesia.

[19] Stoneham MD, Cooper R, Quiney NF, Walters FJ (1996). Pain following craniotomy: a preliminary study comparing PCA morphine with intramuscular codeine phosphate. Anaesthesia.

[20] Ho IHT, Liu X, Zou Y, Liu T, Hu W, Chan H (2019). A Novel Peptide Interfering with proBDNF-Sortilin Interaction Alleviates Chronic Inflammatory Pain. Theranostics.

[21] Ali ZS, Flanders TM, Ozturk AK, Malhotra NR, Leszinsky L, McShane BJ (2019). Enhanced recovery after elective spinal and peripheral nerve surgery: pilot study from a single institution. Journal of neurosurgery Spine.

[22] Ali ZS, Ma TS, Ozturk AK, Malhotra NR, Schuster JM, Marcotte PJ (2018). Pre-optimization of spinal surgery patients: Development of a neurosurgical enhanced recovery after surgery (ERAS) protocol. Clinical neurology and neurosurgery.

[23] Vacas S, Van de Wiele B (2017). Designing a pain management protocol for craniotomy: A narrative review and consideration of promising practices. Surgical neurology international.

[24] Gustafsson UO, Scott MJ, Hubner M, Nygren J, Demartines N, Francis N (2019). Guidelines for Perioperative Care in Elective Colorectal Surgery: Enhanced Recovery After Surgery (ERAS((R))) Society Recommendations: 2018. World journal of surgery.

[25] Patel SY, Garcia Getting RE, Alford B, Hussein K, Schaible BJ, Boulware D (2018). Improved Outcomes of Enhanced Recovery After Surgery (ERAS) Protocol for Radical Cystectomy with Addition of a Multidisciplinary Care Process in a US Comprehensive Cancer Care Center. World journal of surgery.

[26] Parrish AB, O'Neill SM, Crain SR, Russell TA, Sonthalia DK, Nguyen VT (2018). An Enhanced Recovery After Surgery (ERAS) Protocol for Ambulatory Anorectal Surgery Reduced Postoperative Pain and Unplanned Returns to Care After Discharge. World journal of surgery.

[27] Kjolhede P, Bergdahl O, Borendal Wodlin N, Nilsson L (2019). Effect of intrathecal morphine and epidural analgesia on postoperative recovery after abdominal surgery for gynecologic malignancy: an open-label randomised trial. BMJ open.

[28] Kasten BB, Udayakumar N, Leavenworth JW, Wu AM, Lapi SE, McConathy JE (2019). Current and Future Imaging Methods for Evaluating Response to Immunotherapy in Neuro-Oncology. Theranostics.

[29] Liu B, Liu S, Wang Y, Zhao B, Zhao T, Zhao L (2019). Neurosurgical enhanced recovery after surgery (ERAS) programme for elective craniotomies: are patients satisfied with their experiences? A quantitative and qualitative analysis. BMJ open.

[30] Lu D, Wang Y, Zhao T, Liu B, Ye L, Zhao L (2020). Successful implementation of an enhanced recovery after surgery (ERAS) protocol reduces nausea and vomiting after infratentorial craniotomy for tumour resection: a randomized controlled trial. BMC neurology.

[31] Haldar R, Kaushal A, Gupta D, Srivastava S, Singh PK (2015). Pain following craniotomy: reassessment of the available options. BioMed research international.

[32] Dilmen OK, Akcil EF, Tunali Y, Karabulut ES, Bahar M, Altindas F (2016). Postoperative analgesia for supratentorial craniotomy. Clinical neurology and neurosurgery.

[33] Rahimi SY, Alleyne CH, Vernier E, Witcher MR, Vender JR (2010). Postoperative pain management with tramadol after craniotomy: evaluation and cost analysis. Journal of neurosurgery.

[34] Peon AU, Diccini S (2005). [Postoperative pain in craniotomy]. Revista latino-americana de enfermagem.

[35] Gottschalk A, Berkow LC, Stevens RD, Mirski M, Thompson RE, White ED (2007). Prospective evaluation of pain and analgesic use following major elective intracranial surgery. Journal of neurosurgery.

[36] Mordhorst C, Latz B, Kerz T, Wisser G, Schmidt A, Schneider A (2010). Prospective assessment of postoperative pain after craniotomy. Journal of neurosurgical anesthesiology.

[37] Wannemuehler TJ, Rubel KE, Hendricks BK, Ting JY, Payner TD, Shah MV (2016). Outcomes in transcranial microsurgery versus extended endoscopic endonasal approach for primary resection of adult craniopharyngiomas. Neurosurgical focus.

[38] Lau D, Ziewacz JE, Siddiqi HK, Pelly A, Sullivan SE, El-Sayed AM (2012). Cigarette smoking: a risk factor for postoperative morbidity and 1-year mortality following craniotomy for tumor resection. Journal of neurosurgery.

[39] Jia Y, Zhao C, Ren H, Wang T, Luo F (2019). Pre-emptive scalp infiltration with dexamethasone plus ropivacaine for postoperative pain after craniotomy: a protocol for a prospective, randomized controlled trial. Journal of pain research.

[40] Batoz H, Verdonck O, Pellerin C, Roux G, Maurette P (2009). The analgesic properties of scalp infiltrations with ropivacaine after intracranial tumoral resection. Anesthesia and analgesia.

[41] Song J, Li L, Yu P, Gao T, Liu K (2015). Preemptive scalp infiltration with 0.5% ropivacaine and 1% lidocaine reduces postoperative pain after craniotomy. Acta neurochirurgica.

[42] Saringcarinkul A, Boonsri S (2008). Effect of scalp infiltration on postoperative pain relief in elective supratentorial craniotomy with 0.5% bupivacaine with adrenaline 1:400,000. Journal of the Medical Association of Thailand = Chotmaihet thangphaet.

[43] Basuni AS, Ezz HA, Albirmawy OA (2013). Preoperative peritonsillar infiltration of dexamethasone and levobupivacaine reduces pediatric post-tonsillectomy pain: a double-blind prospective randomized clinical trial. Journal of anesthesia.

[44] Guilfoyle MR, Helmy A, Duane D, Hutchinson PJ (2013). Regional scalp block for postcraniotomy analgesia: a systematic review and meta-analysis. Anesthesia and analgesia.

[45] Ryu CM, Yu HY, Lee HY, Shin JH, Lee S, Ju H (2018). Longitudinal intravital imaging of transplanted mesenchymal stem cells elucidates their functional integration and therapeutic potency in an animal model of interstitial cystitis/bladder pain syndrome. Theranostics.

[46] Cata JP, Bhavsar S, Hagan KB, Arunkumar R, Shi T, Grasu R (2018). Scalp blocks for brain tumor craniotomies: A retrospective survival analysis of a propensity match cohort of patients. Journal of clinical neuroscience: official journal of the Neurosurgical Society of Australasia.

[47] Sudheer PS, Logan SW, Terblanche C, Ateleanu B, Hall JE (2007). Comparison of the analgesic efficacy and respiratory effects of morphine, tramadol and codeine after craniotomy. Anaesthesia.

[48] McNicol ED, Ferguson MC, Hudcova J (2015). Patient controlled opioid analgesia versus non-patient controlled opioid analgesia for postoperative pain. The Cochrane database of systematic reviews.

[49] Sarin A, Litonius ES, Naidu R, Yost CS, Varma MG, Chen LL (2016). Successful implementation of an Enhanced Recovery After Surgery program shortens length of stay and improves postoperative pain, and bowel and bladder function after colorectal surgery. BMC anesthesiology.

[50] Rakhman E, Shmain D, White I, Ekstein MP, Kollender Y, Chazan S (2011). Repeated and escalating preoperative subanesthetic doses of ketamine for postoperative pain control in patients undergoing tumor resection: a randomized, placebo-controlled, double-blind trial. Clinical therapeutics.

[51] Urban MK, Ya Deau JT, Wukovits B, Lipnitsky JY (2008). Ketamine as an adjunct to postoperative pain management in opioid tolerant patients after spinal fusions: a prospective randomized trial. HSS journal: the musculoskeletal journal of Hospital for Special Surgery.

[52] McNicol ED, Schumann R, Haroutounian S (2014). A systematic review and meta-analysis of ketamine for the prevention of persistent post-surgical pain. Acta anaesthesiologica Scandinavica.

[53] Lavand'homme P, De Kock M, Waterloos H (2005). Intraoperative epidural analgesia combined with ketamine provides effective preventive analgesia in patients undergoing major digestive surgery. Anesthesiology.

[54] Maessen JM, Dejong CH, Kessels AG, von Meyenfeldt MF, Enhanced Recovery After Surgery G (2008). Length of stay: an inappropriate readout of the success of enhanced recovery programs. World journal of surgery.

[55] Epstein AM, Stern RS, Tognetti J, Begg CB, Hartley RM, Cumella E Jr (1988). The association of patients' socioeconomic characteristics with the length of hospital stay and hospital charges within diagnosis-related groups. The New England journal of medicine.

[56] Woodworth L, Romano PS, Holmes JF (2017). Does Insurance Status Influence a Patient's Hospital Charge?. Applied health economics and health policy.

[57] Liu B, Wang Y, Liu S, Zhao T, Zhao B, Jiang X (2019). A randomized controlled study of preoperative oral carbohydrate loading versus fasting in patients undergoing elective craniotomy. Clinical nutrition.

[58] Yuill KA, Richardson RA, Davidson HI, Garden OJ, Parks RW (2005). The administration of an oral carbohydrate-containing fluid prior to major elective upper-gastrointestinal surgery preserves skeletal muscle mass postoperatively-a randomised clinical trial. Clinical nutrition.

[59] Liu B, Wang Y, Liu S, Zhao T, Zhao B, Jiang X (2018). A randomized controlled study of preoperative oral carbohydrate loading versus fasting in patients undergoing elective craniotomy. Clinical nutrition.

[60] Smith MD, McCall J, Plank L, Herbison GP, Soop M, Nygren J (2014). Preoperative carbohydrate treatment for enhancing recovery after elective surgery. The Cochrane database of systematic reviews.

