# Supplementary material for: Management of Postoperative Pain after Elective Craniotomy: A Prospective Randomized Controlled Trial of a Neurosurgical Enhanced Recovery after Surgery (ERAS) Program
Source: Int J Med Sci. 2020 Jun 21;17(11):1541–9. doi: 10.7150/ijms.46403 (PMC7359387; doi:10.7150/ijms.46403)
Supplement: Supplementary file 1 — Supplementary figures and tables. [file ijmsv17p1541s1.pdf]

Supplementary Information

Supplementary file 1. Research Protocol
Supplementary file 2. Neurosurgical ERAS record checklist
Supplementary file 3. ERAS Protocol For Elective Craniotomies

Supplementary file 1. Research Protocol

Project summary

Although ERAS programs have gained increasing acceptance in various surgical specialties, there is currently no established neurosurgical ERAS protocol for patients undergoing elective craniotomy reported in literature. Here, we try to evaluate the design, implementation, safety and efficacy of a novel neurosurgical enhanced recovery after surgery (ERAS) protocol for elective craniotomy in a tertiary center located in China. A multi-disciplinary neurosurgical ERAS protocol for elective craniotomy was developed, based on the best available evidence. A total of 300 patients undergoing elective craniotomy between Oct 2016 and Nov 2017 were enrolled in a randomized clinical trial (RCT) comparing our novel ERAS protocol to conventional neurosurgical perioperative management. The primary end point was the evaluation of postoperative pain by means of a verbal numerical rating scale (NRS). Secondary end points included Secondary outcome measures included Median total hospital length of stay from admission to discharge, Median post procedure length of stay from end of procedure to discharge， Readmission rate 30 day all cause readmission rate， Reoperation rate reoperation for any indication within 30 days and Total cost of hospitalization (RMB). This multidisciplinary, evidence-based neurosurgical ERAS protocol for craniotomy appears to have significant benefits compared to conventional perioperative management. Implementation of a neurosurgical ERAS protocol for elective craniotomies, which resulted in alleviating postoperative pain and enhancing recovery after surgery.

General information

- Public title： Clinical study on the development and efficacy evaluation of Enhanced Recovery After Surgery (ERAS) in Neurosurgery
- Registration number：ChiCTR-INR-16009662
- Date of Registration： 2016-10-27
- Date of approved by ethic committee： 2016-10-25
- Name of the ethic committee：Ethical committee of Tangdu Hospital，Fourth Military Medical University
- Primary sponsor： Department of neurosurgery, Tangdu Hospital, Fourth Military Medical University
- Primary sponsor's address： 569 Xinsi Rd, Baqiao District, Xi'an, Shaanxi, China

- Source(s) of funding：China national health and Family Planning Commission
- Study leader： Shiming He,MD., PhD.

  Phone: +86 29 84717737

  Fax: +86 29 84717737

  Email: he-shiming@163.com
- Study design： Randomized parallel controlled trial

Rationale & background information

Conventional craniotomy is typically associated with significant physiologic stressors and prolonged functional recovery. An excessive stress response may predispose patients to an increased risk of cardiovascular and cerebrovascular complications, nutrient malabsorption and delayed convalescence[1]. With the increasing understanding of perioperative pathophysiology, the concept of enhanced recovery after surgery (ERAS), originally introduced by Kehlet in 1997, has been established in an effort to improve functional outcomes after surgery and decrease perioperative morbidity[2, 3]. Several ERAS protocols have gained acceptance in a wide variety of surgical subspecialties[4-8]. However, to the best of our knowledge, ERAS protocols within neurosurgery, specifically for elective craniotomy, have not been established. Owing to the rapid development of neurosurgery in recent decades worldwide, minimally invasive craniotomy have benefited huge numbers of patients with improved patient recovery and satisfaction[9]. Based on the core concept of evidence-based review of ERAS and ERAS protocols for abdominal and pelvic surgeries, Hagan el al. proposed a preliminary set of recommendations including seventeen ERAS items for creating a standardized protocol for craniotomy[10]. However, the safety and feasibility of implementing a detailed neurosurgical ERAS protocol for craniotomy in a clinical setting has not been previously described in the literature. Here, we describe our experience with the implementation of a novel, multi-disciplinary, evidence-based neurosurgical ERAS protocol for elective craniotomy at a large tertiary hospital in China.

Study goals and objectives

The aim of this study was to prospectively evaluate the efficacy of improvement on postoperative pain after elective craniotomies based on neurosurgical enhanced recovery after surgery (ERAS) protocol for elective craniotomies.

Study Design

Study type：Interventional study

Study phase：New Treatment Measure Clinical Study

Study design：Randomized parallel controlled trial

Inclusion criteria:

(1) Patients with single intracranial lesion and medically eligible for elective craniotomy;

(2) Age between 18-65 years;

(3) Patients who are able to communicate well with the medical staff;

(4) Patients who understand and sign the Informed Consent, with good compliance in the study.

Exclusion criteria：

(1) non-brain tumor patients, such as severe craniocerebral injury leading to bilateral mydriasis, vital signs were not stable;

(2) children (patients less than 18 years), awake craniotomy;

(3) patients with severe spinal cord injury shock;
(4) other trauma caused by preoperative cardiac arrest, combined with severe limb fractures or thoracic and abdominal injury;
(5) infection or inflammation in the surgical area;
(6) serious complications of disease (blood system, respiratory system, digestive system, etc.) patients;
(7) patients with severe heart disease (such as coronary heart disease, myocardial infarction, etc.);
(8) Patients with ULN and / or renal function (Cr)> 1.5 times ULN with liver function (ALT, AST)> 2 times;
(9) patients with mental illness;
(10) Women who have a childcare plan within 6 months of pregnancy or breastfeeding;
(11) Other patients who were considered unsuitable for inclusion in the study.

Methodology

Interventions：

- Group： ERAS Group

  Intervention： Perioperative ERAS protocol for Neurosurgery Sample size：120

- Group： Control Group

  Intervention：Conventional neurosurgery perioperative management Sample size：120

Countries of recruitment and research settings：

Country： China Province： Shaanxi City： Xi'an

Institution hospital： Tangdu Hospital Level of the institution： Tertiary A hospital

Flow Diagram

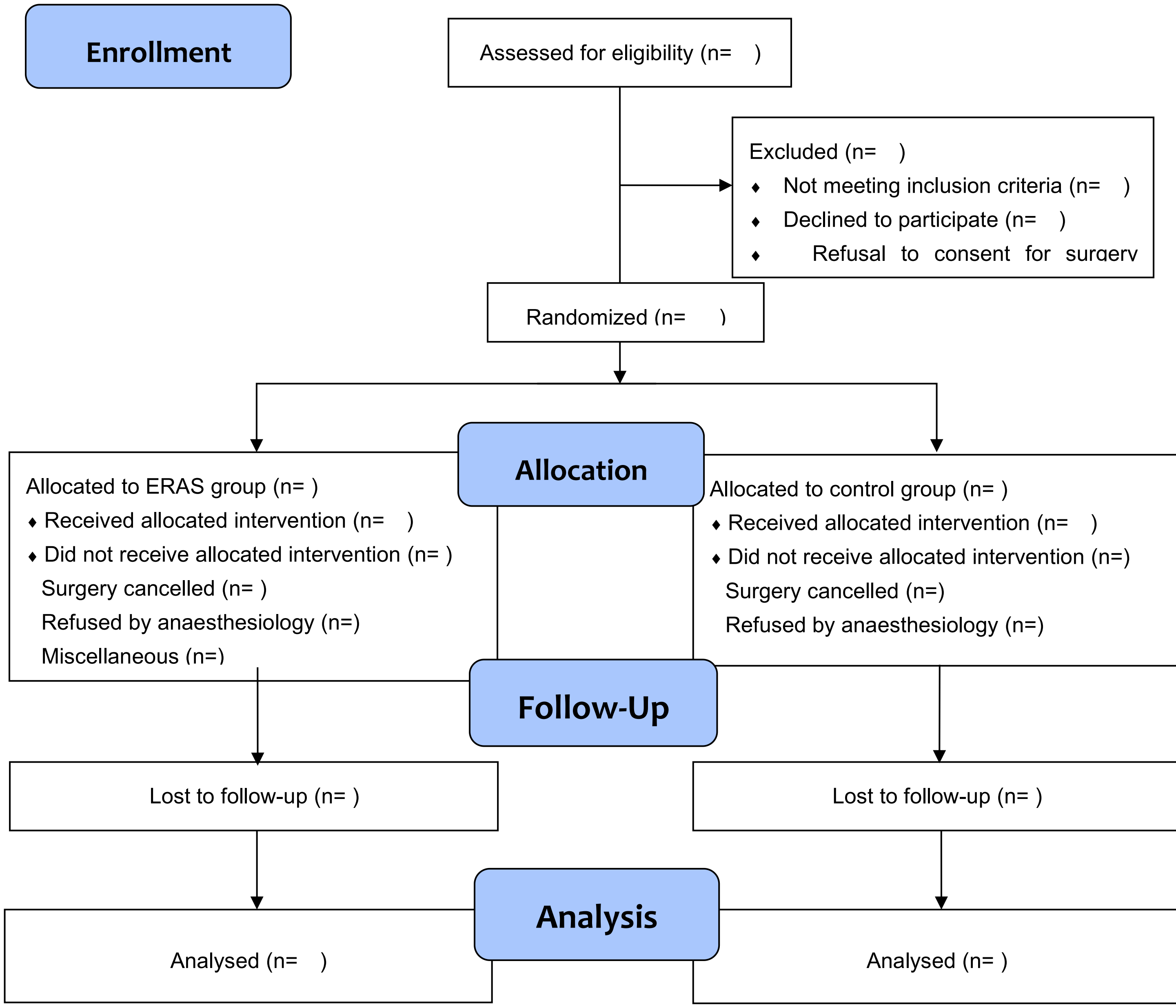

From Oct 2016 to Nov 2017, patients aged from 18 to 65 years, who were admitted for elective craniotomies at Department of Neurosurgery, Tangdu Hospital were enrolled for this study. After obtaining informed consents, patients were prospectively randomized into two groups by simple randomization procedures (computerized random numbers) by the research coordinator. Due to the requirement for active patient participation, it was not possible to perform the study with blinded participants and care providers. Only those who collected and assessed outcomes were blinded.

We were supported by the local institutional ethnical committee to develop a neurosurgical ERAS protocol through a quality patient care initiative. Institutional review board (IRB) approval was also obtained prior to consenting patients for this study. In June 2016, we set up a Neurosurgical ERAS Working Group, including clinicians and ancillary staff from neurosurgery, anesthesiology, in-patient and operative nursing, as well as nutrition services. This multidisciplinary working group was then used to develop and apply the neurosurgical ERAS protocol outlined in this study. The protocol was designed for patients undergoing elective craniotomy, and adapted from concepts elicited from other established protocols for general surgery and was done after an extensive review of the current evidence-based perioperative care interventions supported in the literature. However, some critical concepts for abdominal and/or pelvic surgery do not apply to neurosurgical patients, and were thus excluded from our protocol. In addition, we reviewed the published literature on other successful ERAS protocols, particularly the preliminary ERAS recommendations for oncological craniotomy proposed by Hagan el al[10].

Outcome measurements:

Data were collected from patient demographic data (age, sex), preoperative nutritional record (total bodyweight, body mass index), preoperative co-morbidity status (American association of anesthesiologists grades, ASA grades) and other presenting physical characteristics (smoking, diabetes, history of post-operative nausea and vomiting (PONV), motion sickness, hypertension, hypercholesterolemia, etc.) were assessed and recorded at admission.

Data of surgical procedures like types of operation, lesion locations (supratentorial superficial lesion, supratentorial deep-seated lesion or infratentorial lesion), length of procedure, blood loss, blood transfusion and intraoperative fluid were assessed.

The primary end point was the evaluation of postoperative pain by means of a verbal NRS ranging from 0 to 10, with 0 representing no pain and 10 representing the worst pain imaginable. Postoperative pain was recorded from 1 day after extubation in the neurosurgical intensive care unit to the day of discharge. The nonopioid analgesic drugs， weak opioid analgesics (+ nonopioid analgesic drugs) and strong opioid analgesics (+ nonopioid analgesic drugs) were administered for postoperative pain treatment depending on the assessment and decision of the attending team.

Secondary outcome measures included median of the total hospital length of stay from admission to discharge, median of post procedure length of stay from end of procedure to discharge, readmission rate within 30 days, reoperation rate within 30 days and total cost of hospitalization (RMB).

Safety Considerations

- Assessment of safety:

  Safety data will be inclusive of all adverse effects (AEs), from the point of subject enrolment to the final follow-up visit or discontinuation, whichever comes first. Reports of AEs will minimally include the following information; date of event; diagnosis or description of the event; assessment of the seriousness; treatment; outcome and date.

- Discharge criteria:

  Patients in this study, either in ERAS or control group, were discharged once they met our predefined discharge criteria, which included: adequate pain management with oral analgesia, adequate intake of solid food, without the need for intravenous fluids, no fever, independent mobility and a safe disposition home. The decision to discharge was made via the consensus of two senior attending physicians in the department of Neurosurgery, who were instructed to follow the discharging criteria, and were independent of the researchers involved in this study.

Follow-Up

Data on patient characteristics, intraoperative parameters and perioperative course were collected during the hospitalization and at the 4 months follow-up.

Data Management and Statistical Analysis

Descriptive statistics of ERAS group and control group were compared for all relevant patient characteristics. To compensate for potential dropouts, patients were enrolled. Interim analysis was planned when the minimal number of the predefined sample size was met. Continuous data with a normal distribution were statistically tested for group differences using chi-square test and Fisher's exact text. Logistic regression and chi-square test were used to assess the potential relationship between incidence and severity of pain and potential influencing factors. The statistical analysis was performed with SPSS program for Windows (Ver. 19, IBM Corp., Armonk, NY). A P value of <0.05 was considered to be statistically significant.

Expected Outcomes of the Study

This multidisciplinary evidence-based neurosurgical ERAS protocol for elective craniotomies appears to have significant benefits compared to the conventional care. Implementation of a neurosurgical ERAS protocol for elective craniotomies, which resulted in alleviating postoperative pain and enhancing recovery after surgery.

Dissemination of Results and Publication Policy

Final study results and conclusions will be presented at international conferences and publications in peer-reviewed journals.

Duration of the Project: From Oct 2016 to Nov 2017

Problems Anticipated

First, the subgroup analysis was need to perform with all consecutive patients within the ERAS pathway and conventional surgery protocol. Postoperative pain management is embedded in a multidisciplinary cooperation and the impact of pain management on recovery, pain relief, and length of stay needs to be interpreted in this context. Second, little information was known regarding the individual contribution of the interventions, which may be investigated in further studies. As mentioned in the Methods section, the enhanced recovery pathway was adapted during

the study period. To avoid the bias of various perioperative care pathways and unbalanced interventions.

Ethics

Informed consent was obtained from all individual participants or their legal representatives included in this study. The analysis and usage of patient information for this study was approved by the Ethical Committee of Tangdu Hospital. And the methods were carried out in accordance with the approved guidelines. This randomized control trial (RCT) was registered at Chinese Clinical Trial Registry (Registration date: October 27, 2016, http://www.chictr.org.cn/showproj.aspx?proj=16480) with registration number ChiCTR-INR-16009662.

Informed Consent Forms

The approved version of the protocol must have copies of informed consent forms (ICF), both in English and the local language in which they are going to be administered.

References

1. Ren L, Zhu D, Wei Y, Pan X, Liang L, Xu J, et al. Enhanced Recovery After Surgery (ERAS) program attenuates stress and accelerates recovery in patients after radical resection for colorectal cancer: a prospective randomized controlled trial. World J Surg. 2012; 36: 407-14.
2. Kehlet H, Wilmore DW. Multimodal strategies to improve surgical outcome. American journal of surgery. 2002; 183: 630-41.
3. Ljungqvist O, Scott M, Fearon KC. Enhanced Recovery After Surgery: A Review. JAMA surgery. 2017; 152: 292-8.
4. Cerantola Y, Valerio M, Persson B, Jichlinski P, Ljungqvist O, Hubner M, et al. Guidelines for perioperative care after radical cystectomy for bladder cancer: Enhanced Recovery After Surgery (ERAS((R))) society recommendations. Clinical nutrition. 2013; 32: 879-87.
5. Melloul E, Hubner M, Scott M, Snowden C, Prentis J, Dejong CH, et al. Guidelines for Perioperative Care for Liver Surgery: Enhanced Recovery After Surgery (ERAS) Society Recommendations. World J Surg. 2016; 40: 2425-40.
6. Scott MJ, Baldini G, Fearon KC, Feldheiser A, Feldman LS, Gan TJ, et al. Enhanced Recovery After Surgery (ERAS) for gastrointestinal surgery, part 1: pathophysiological considerations. Acta anaesthesiologica Scandinavica. 2015; 59: 1212-31.
7. Mortensen K, Nilsson M, Slim K, Schafer M, Mariette C, Braga M, et al. Consensus guidelines for enhanced recovery after gastrectomy: Enhanced Recovery After Surgery (ERAS(R)) Society recommendations. The British journal of surgery. 2014; 101: 1209-29.
8. Thorell A, MacCormick AD, Awad S, Reynolds N, Roulin D, Demartines N, et al. Guidelines for Perioperative Care in Bariatric Surgery: Enhanced Recovery After Surgery (ERAS) Society Recommendations. World J Surg. 2016; 40: 2065-83.
9. Garrett M, Consiglieri G, Nakaji P. Transcranial minimally invasive neurosurgery for tumors. Neurosurgery clinics of North America. 2010; 21: 595-605, v.
10. Hagan KB, Bhavsar S, Raza SM, Arnold B, Arunkumar R, Dang A, et al. Enhanced recovery after surgery for oncological craniotomies. Journal of clinical neuroscience : official journal of the Neurosurgical Society of Australasia. 2016; 24: 10-6.

Supplementary file 2. Neurosurgical ERAS record checklist

Name		Hospital ID		Reg. No.	
Sex		Age		Admission date	Year/month/day
Contact person		Phone		Home address	
Doctor-in-charge		Nurse-in-charge	/	Diagnosis	
Anesthesiologist		Date of operation	Year/month/day	Operation	

ITEMS		EXECUTION
Preoperative evaluation	Inform consent	YES□ NO□
	Epilepsy	NO□ YES□ → medicine: ___________
	Nrs2002	Total（ ）= Disease（ ）+ Nutritional（ ）+ Age（ ）
	Nutritional status	Height:____(Cm) Weight:______(Kg)<br>BMI:____(Kg/M²) Body Fat:_____(Kg)<br>Lean Muscle Mass:_____(Kg) Grip Strength:______(Kg)
	SGA score	A□ B□ C□
	KPS score	100□ 90-80□ 80-70□ 70-60□ 60-50□ (______)
	HAD anxiety	0-7□ 8-10□ 11-20□ (______)
	HAD depression	0-7□ 8-10□ 11-20□ (______)
	Pain intensity	1-3□ 4-6□ 7-9□ 10□ (______)
	Autar DVT risk	≤6□ 7-10□ 11-14□ ≥15□ (______)
	Caprini risk	0-1□ 2□ 3-4□ 5□
	PONV risk score	0□ 1□ 2□ 3□ 4□
	Pressure ulcer risk assessment	≤10□ 11-12□ ≥13□ (______)
	Asa grade	I□ II□ III□ IV□ V□
Preoperative nursing	Abstinence from alcohol & smoking	YES□ NO□
	Respiratory training	NO□ YES□ →<br>Methods: blowing balloons□ walking & mild climbing□
	Mouth-breathing exercise	YES□ NO□
	On-bed urinary & excretion exercise	YES□ NO□

	Pre-op last defecation time	Operational day morning□<br>Pre-op: 1d□ 2d□ >2d□	
	Pre-op intestinal intervention	NO□ YES□<br>→ Cleansing Enema☐ Glycerol Enema☐ Others: ________	
Preoperative nursing	Area of skin preservation	Total Shaving☐ Partial Shaving☐<br>2cm Shaving around the incision☐	
Preoperative nursing	Hair cleaning	YES□ NO□	
Preoperative nursing	Gargle/nasal drops usage	NO□ YES□ → for______days	
Preoperative nursing	pre-op pulmonary protection	NO□ YES□ → Ambroxol☐ Budesonide☐	
Preoperative nursing	Fasting solid food	6h□ 8h□ 10h□ 12h□	
Preoperative nursing	Intake nutrient solution	2h□ 4h□ 6h□ 8h□ NO□	Preoperative fasting<br>Glucose level: _______ mmol/l
Preoperative nursing	Intake glucose liquid	2h□ 4h□ 6h□ 8h□ NO□	Glucose level prior<br>To the or:______ mmol/l
Preoperative nursing	Last pre-op liquid food	4h□ 6h□ 8h□	
Preoperative nursing	Pre-op nausea & vomiting	NO□ YES□ → score:______ medicine and dose:________	
Operating Room Nursing	OR parameters	Room Temperature:____℃ Humidity:____%	
Operating Room Nursing	OR stay time	Entry Time:__Hour : Min__; Leave Time: Hour : Min ; Total: Hour : Min__	
Operating Room Nursing	Surgical time	Begin:__Hour : Min__ Finish:__Hour : Min__ Total:__Hour : Min__	
Operating Room Nursing	Entry conscious state	Sober☐ Somnolence☐ Lethargy☐ Light Coma☐	
Operating Room Nursing	Psychological status	Nervous☐ Anxiety☐ Calm☐ Indifferent☐	
Operating Room Nursing	Urinary catheterization	Post general anesthesia□<br>Before general anesthesia□	
Operating Room Nursing	Antibiotic prophylaxis	Medicine:__________ Dose:________<br>Additional dose: YES□ NO□	
Operating Room Nursing	Transfusion reaction	□NO adverse transfusion reactions □YES, **†Special Case Records**	
Operating Room Nursing – Avoiding hypothermia	Body temp	Entry temp: ℃ Maintain to:<36℃□ 36-37℃□ >37℃□	
Operating Room Nursing – Avoiding hypothermia	Heating pad	YES□ NO□ heated to ___℃	
Operating Room Nursing – Avoiding hypothermia	Intravenous fluid administration	YES□ NO□ heated to ___℃	
Operating Room Nursing – Avoiding hypothermia	Flushing fluid	YES□ NO□ heated to ___℃	
Operating Room Nursing – VTE prevent	Measures	Compression Stocking☐ Pneumatic Pump☐ ☐Medication:	
Operating Room Nursing – VTE prevent	Intra-op	Supine Position☐ Lateral Position☐（Left☐ Right☐）	

		position	Prone Position☐ Sitting Position☐ Others:
		Pressure area care	☐Head (Occipital. Ears, Eyes) ☐The Trunk (Shoulder. Iliac Spine. Sacral Tail) ☐Limbs (Elbows, Knees, Heels. Toes)
		Care frequency	1-2□ 3-4□ 5-6□ 7-8□ 9-10□ >10□
**Anesthesia management**	Anesthesia time		Begin:__Hour : Min__ Finish:__Hour : Min__ Total:__Hour : Min__
	Types of anesthesia		Intravenous☐ Inhalational ☐ Anesthesia☐
	Monitored parameters		EEG☐ Cardiac Output☐ Muscle Relaxants☐<br>Body Temp☐ Arterial Blood Gases Analysis☐
	Pre-op scalp incision anesthesia		NO□ YES□ → Medicine: Ropivacaine☐ Others:
	Post-op scalp incision anesthesia		NO□ YES□ → Medicine: Ropivacaine☐ Others:
	Intraoperative circulation		□Stable ☐Low Perfusion Duration:____min<br>(**†Special Case Records**)
	liquid discharging		Blood loss:_______ml urinary volume:_______ml total:_______ml
	Liquid loading		Crystalloid solution:_______ml Colloidal solution:_______ml<br>Total:_______ml
	Transfused blood		□NO □YES: Erythrocyte_____U, Plasma______ml, Cryo_____U
	Transfusion reaction		□NO □YES **†Special Case Records**
	Consciousness		0□ 1□ 2□ 3□ 4□
	Steward grade		Total（ ）= Consciousness（ ）+ Breath（ ）+ Body Movement（ ）
	Extubation before leaving OR		YES□ breathing without extubation□ NO breath□
**Surgical manipulations**	Pre-op mucosal protection		□NO □YES → medicine:_______
	Drainage tube		□NO □YES → Quantity: _______<br>Position:□Surgical Field ☐Epidural ☐EVD
	Drainage removal		<24h□ 24-48h□ >48h□
	Duraplasty		Absorbable suture□ Non-absorbable suture□ （Tight☐ Un-Tight□）
	Subcutaneous suture		Absorbable suture□<br>Non-Absorbable suture☐ (Intermittent stitching☐ Continuous stitching☐)
	Skin suture		Absorbable suture□<br>Non-Absorbable Suture☐ Stapler☐ （Intradermal☐ Intermittent☐ ）
**Postoperative management**	Postoperative pain management	Pain location	□Surgical Area ☐Head ☐Other:
		Characteristic of pain	Dull☐ Stinging☐ Swelling Pain☐<br>Compressive☐ （Intermittent☐ Persistence☐）
		Numeric rating scales (NRS)	POD 1 | 1-3□ 4-6□ 7-9□ 10□
			POD 2 | 1-3□ 4-6□ 7-9□ 10□
			POD 3 | 1-3□ 4-6□ 7-9□ 10□
			POD 4 | 1-3□ 4-6□ 7-9□ 10□

		Duration	1-2d□ 2-3d□ 3-4d□
		Analgesics	□NO □YES: Phase 1☐ Phase 1☐ Phase 1□ medicine:_____
		PCA	□NO □YES medicine:_____
	Respiratory management		Atomization:□NO □YES medicine:_____<br>Intravenous infusion: □NO □YES medicine:_____
	Epilepsy prevention		□NO □YES: Sodium Valproate☐ Oxcarbazepine☐<br>Phenobarbital☐ Others☐:_____
	Digestive system management	Mucosa protection	Omeprazole☐ Esomeprazole☐ Others□:____
		Vomiting prevention	□NO □YES medicine:_____
		Nausea grade	□NO ☐Slight ☐Mild ☐Severe
		Vomit grade	Grade 0☐ Grade 1☐ Grade 2☐ Grade 3□
		PONV VAS score	1～4□ 5～6□ 7～10□
		Antiemetic drug	□NO □YES medicine: ____
	Re-examination CT scan		Good☐ Hematoma☐ Edema☐ Infarction☐<br>Detailed Description: ________________________________
	Post-op complications		NO□ YES□: Complications:
	Daily liquid loading		POD1: ______ml; POD2:_______ml; POD3:_______ml; POD4:_______ml
	Wound healing		A□ B□ C□
**Postoperative Nursing Management**	Extubating time		Already extubated□ 0-2h□ 2-4h□ 4-6h□ >6h□
	Time for removing urinary catheter		6h□ <24h□ >24h□
	Prophylactic thrombosis		Compression stocking □NO □YES<br>Pneumatic pump □NO □YES Limb movement □NO □YES
	Post-op Braden score		>18□ 18-15□ 14～13□ 12～10□ ≤9□
	On-bed exercise		Immediately after awaken□ 4-8h after awaken□ >8h after awaken□
	Ambulation		1d□ 2d□ 3d□ 4d□
	Intake water		4h□ 6h□ 8h□ 12h□ 24h□ >24h□
	Intake nutrient solution		6h□ 8h□ 12h□ 24h□ >24h□
	Intake liquid food		6h□ 8h□ 10h□ 12h□ 24h□ 36h□
	Intake solid food		24h□ 24-48h□ >48h□
	Parenteral nutrition		□NO □YES medicine:
	Post-op i.v. stop time		2d□ 3d□ 4d□ 5d□ >5d□

	Post-op complications	Pulmonary infection:□NO □YES DVT:□NO □YES<br>Epilepsy:□NO □YES Gastrointestinal bleeding:□NO □YES<br>Transfusion reaction:□NO □YES **(†Special Case Records)**
**Discharge Evaluation**	Nutritional status	Height:____(Cm) Weight:___(Kg)<br>BMI:____(Kg/M²) Body Fat:__(Kg)<br>Lean Muscle Mass:____(Kg) Grip Strength:____(Kg)
	SGA score	A□ B□ C□
	Mental state	Good☐ Fair☐ Poor☐ Others☐:_____
	HAD anxiety	0-7□ 8-10□ 11-20□ ( )
	HAD depression	0-7□ 8-10□ 11-20□ ( )
	KPS score	100□ 90-80□ 80-70□ 70-60□ 60-50□ 50-30□ ( )
	Patient satisfaction	>90□ 80-90□ <80□ ( )
	Hospitalization	Total cost of hospitalization: RMB
		Length of stay:_______days
		Post-operative length of stay:_______days
**Follow-Up**	1st Follow-up time	2 Weeks☐ 4 Weeks☐ Others:
	1st Follow-up method	Telephone☐ Office Visit☐ Others☐:
	1st KPS score	100□ 90-80□ 80-70□ 70-60□ 60-50□ 50-30□ ( )
	2nd Follow-up time	2 Weeks☐ 4 Weeks☐ Others:
	2nd Follow-up method	Telephone☐ Office Visit☐ Others☐:
	2nd KPS score	100□ 90-80□ 80-70□ 70-60□ 60-50□ 50-30□ ( )

Supplementary file 3. ERAS Protocol For Elective Craniotomies

Phase	Items	Control group		ERAS group		ERAS recommendation elements
		Content	Procedures	Content	Procedures	
**Admission**	Evaluation	Based on the inclusion criteria and random number, patients were enrolled in the Control group.	Sign the Informed Consent on ERAS for neurosurgery	Based on the inclusion criteria and random number, patients were enrolled in the ERAS group.	Sign the Informed Consent on ERAS for neurosurgery	
**Pre-operational evaluation**	Preoperative counseling	Outpatient and pre-hospital consultation		Outpatient and pre-hospital consultation		√ Preoperative counseling
	Preoperative functional status evaluation	Pre-operative KPS score		Pre-operative KPS score	Preoperative KPS assessment	
	Preoperative smoking and alcohol consumption	Abstinence from both alcohol and smoking at least for 2 weeks		Abstinence from both alcohol and smoking at least for 2 weeks	Quit smoking and drinking	√ Preoperative smoking and alcohol consumption
	Mental state assessment	Anxiety and depression evaluation	Hospital Anxiety and Depression Scale (HADS)	Anxiety and depression evaluation	Hospital Anxiety and Depression Scale (HADS)	
	Nutritional assessment	NRS2002, nutritional status assessment	Administration of nutritional therapy if necessary	NRS2002, nutritional status assessment, PG-SGA	Administration of nutritional therapy if necessary	√ Preoperative enteral nutrition and perioperative oral immune nutrition
	Evaluation and prophylactic antithrombotic therapy	Based on VTE Caprini Risk Assessment Scale & Autar DVT Risk Scale	1. Lower limbs active/passive activity<br>2. Lower limbs with graduated compression stockings<br>3. intermittent pneumatic compression pump treatment	VTE Caprini Risk Assessment & Autar DVT Risk Assessment Scale	1. Lower limbs active/passive activity<br>2. Lower limbs with graduated compression stockings<br>3. Intermittent pneumatic compression pump treatment	√ Anti-thrombotic prophylaxis
	PONV risk score	PONV Simple Risk Assessment Scale	No prophylaxis	PONV Simple Risk Assessment Scale	Prophylaxis: Score ≥3, preventive vomiting treatment, dexamethasone, 5-HT receptor antagonist (tropisetron)	√ PONV
**Preoperative preparation**	Preoperative intestinal intervention	No	No	Defecation condition	Glycerine Enema induction if long history of constipation or ≥ 2 days without defecation	

Supplementary file 3. ERAS Protocol For Elective Craniotomies

Phase	Items	Control group		ERAS group		ERAS recommendation elements
		Content	Procedures	Content	Procedures	
	Antimicrobial prophylaxis and skin preparation	Routine scalp shaving	Neurosurgeon's preference	Minimize scalp shaving	1. Washing hair with chlorhexidine<br>2. Routine prophylaxis with cefazolin within 1 hour prior to skin incision<br>3. Shaving 1.5-2 cm beyond the margin of the incision.	√ Antimicrobial prophylaxis and skin preparation
	Oral and nasal cavity preparation	No	No	Mouthwash & nasal drops	Apply mouthwash and nasal drops	
	Preoperative water fasting	Routine fasting water for 4 hours, fasting food for 6-8 hours	Follow the routine surgical procedure and surgeon's discretion	Preoperative 2-6 hours oral maltodextrin fructose solution (400 ml)	Fasting solid food for 6 hours<br>Oral intake maltodextrin fructose solution (400 ml) in the morning of operational day	√ Preoperative fasting and carbohydrate loading
	Respiratory intervention	No	Physical exercise: chest movement, balloon blowing, abdominal breathing exercises.	Preoperative respiratory protection	1. Oral and nasal cavity preparation: mouthwash and nasal drops.<br>2. Physical exercise: chest movement, balloon blowing, abdominal breathing exercises, cough training, inspiratory muscle training.<br>3. High risk factor intervention: Age, past and concomitant diseases, estimated surgical time, mucolytics and expectorants	
**The operation day**	Scalp incision anesthesia	No	No	Ropivacaine (0.2%)	1. Subcutaneous local anesthesia before incision and wound suturing.<br>2. Add dose if operational time more than 3h	√ Scalp blocks
	Micro-invasive surgery for craniotomy	Limited in minimally invasive craniotomies, excluding endoscopic skull base approaches.	Follow the minimal invasive neurosurgical procedure and surgeon's discretion	Limited in minimally invasive craniotomies, excluding endoscopic skull base approaches.	Follow the minimal invasive neurosurgical procedure and surgeon's discretion	√ Minimally invasive craniotomies and endoscopic skull base approaches
	Anesthetic protocol	Intravenous-inhalation combined anesthesia	Follow the routine anesthetic procedure.	Intravenous-inhalation combined anesthesia	Follow the institutional routine anesthetic procedure.	√ Anesthetic protocol

Supplementary file 3. ERAS Protocol For Elective Craniotomies

Phase	Items	Control group		ERAS group		ERAS recommendation elements
		Content	Procedures	Content	Procedures	
	Non-opioid analgesia	Opioid analgesia are not usual administrated	1. Follow patient's feedbacks and surgeon's discretion.<br>2. Postoperative morphine and equivalent opioids were not usual prescribed only if the pain VAS≥7 in craniotomy surgeries	Not usual administrated	1. Post-operative pain VAS≥5: Acetaminophen or NSAIDS.<br>2. Postoperative VAS≥7: Central analgesic drugs. Morphine and equivalent opioids.	√ Non-opioid analgesia
	Avoiding hypothermia	Routine	Non-invasive cardiac output monitoring to keep volume status and hemodynamic stability	Measures to prevent hypothermia during the operation	1. Forced-air and electric heating pad<br>2. Warmed liquid for infusion and washing	√ Avoiding hypothermia
	Fluid balance	Restrictive protocol	1. Goal-directed fluid restriction (GDFR) strategy<br>2. Non-invasive cardiac output monitoring to keep volume status and hemodynamic stability	Restrictive protocol and warmed fluids.	1. Goal-directed fluid restriction (GDFR) strategy<br>2. Non-invasive cardiac output monitoring to keep volume status and hemodynamic stability	√ Fluid balance
	Stitching	Routine incision stitching		Dural, subcutaneous tissue and skin are sutured by absorbable suture	Skin is treated with intradermal suture	
	Drainage tube placement	Place drainage tube for most surgeries.		Do not place drainage tube in exception of special circumstances	If the drainage tube is placed, remove it within 48 hours if possible.	
	Prophylactic antibiotics usage	Perioperative prophylactic antibiotics application	1. First dose was given 30 and 60 minutes before the surgery.<br>2. Second dose used if a surgery lasts >4 hours.	Perioperative prophylactic antibiotics application	1. First dose was given 30 and 60 minutes before the surgery.<br>2. Second dose used if a surgery lasts >4 hours.	
	Pain management	Patient-controlled analgesia (PCA)	PCA was given according to anesthetist’s individual preference	Patient-controlled analgesia (PCA)	Placement of PCA with mixed opioids and non-opioids at the end of surgery	

Supplementary file 3. ERAS Protocol For Elective Craniotomies

Phase	Items	Control group		ERAS group		ERAS recommendation elements
		Content	Procedures	Content	Procedures	
**Post-operational management**	Diet	POD 1-2: flow food<br>POD 3: semi-liquid diets<br>POD 4: semi-liquid diets + ordinary diets<br>POD 5: ordinary diets		1. 4 hours after awake: water<br>2. 6-12 hours: half of the nutrient solution (100 ml)<br>3. 12-24 hours: half of the nutrient solution + flow food<br>4. 24-48 hours: half of the nutrient solution + normal diet<br>5. 48 hours after: the ordinary diet	1. POD1: 250-500ml nutrient solution<br>2. POD2: 500-1000ml nutrient solution + ordinary diet	√ Postoperative artificial nutrition
	Pain treatment	Post-operative pain VAS score<br>Step analgesic measures	Score 4-6,NSAIDS<br>Score≥7, Central analgesic drugs	Post-operative pain VAS<br>Step analgesic measures	Score 4-6, acetaminophen or NSAIDS<br>Score≥7, central analgesic drugs	
	Urinary drainage	Used for the duration of the operation and early removal	Removal of the urinary drainage on POD 1-2	Used for the duration of the operation and early removal	Early removal of the urinary drainage within 6h.	√ Urinary drainage
	Respiratory management	Intravenous and atomized medicine administration	1. Expectorant + mucolytics (ambroxol hydrochloride) and/or bronchodilator.<br>2. Glucocorticoids (budesonide) + β2 agonists, inhalants (salbutamol) and/or anticholinergics/muscarinic antagonist (ipratropium bromide).	Intravenous and atomized medicine administration<br>Pharmacologic agents that promote airway clearance	1. Expectorant + mucolytics (ambroxol hydrochloride) and/or bronchodilator.<br>2. Glucocorticoids (budesonide) + β2 agonists, inhalants (salbutamol) and/or anticholinergics/muscarinic antagonist (ipratropium bromide).	
	Digestive system management	Mucosal protection	PPIs (omeprazole, esomeprazole)	Mucosal protection	PPIs (omeprazole, esomeprazole)	
	PONV	PONV VAS	1. No prevention.<br>2. Intervention: dexamethasone, 5-HT receptor antagonist (tropisetron).<br>3. Severe case: droperidol, promethazine	PONV Simple Risk Assessment Scale<br>Prevetion: 3-5 points, preventive anti-vomiting medicine.<br>Intervention: ≥5 points	1. Prevention: PONV VAS≥3, apply dexamethasone, 5-HT receptor antagonist (tropisetron)<br>2. Intervention: PONV VAS≥5, apply 5-HT receptor antagonist (tropisetron) again.<br>3. Severe cases: tropisetron + droperidol, promethazine	√ PONV

Supplementary file 3. ERAS Protocol For Elective Craniotomies

Phase	Items	Control group		ERAS group		ERAS recommendation elements
		Content	Procedures	Content	Procedures	
	Prophylactic antiepileptic drug therapy	Prophylactic AED use is carefully considered	Prophylactic AED use is carefully considered during the perioperative course	Prophylactic antiepileptic drug therapy discouraged.	Insufficient evidence to recommend in favor or against postoperative prophylactic AED discontinuation	
	Daily liquid volume	Routine liquid management	POD 0-1:3000-2000 ml<br>POD 2: 2000 ml	Rapid de-escalation of fluids	POD 0-1: 1000-2000 ml<br>POD 2: 0-1000 ml	
	Post-operational radiological assessment	CT & MRI check after surgery	Perform CT scan on POD 1.<br>Perform MRI scan within 3 days after surgery.	CT & MRI check after surgery	Perform CT scan on POD 1.<br>Perform MRI scan within 3 days after surgery.	
	Preventive antithrombotic therapy	The day after the operation to patient discharge from hospital	1. Lower limbs active/passive activity<br>2. Lower limbs with elastic stockings<br>3. Pneumatic pump treatment	The day after the operation to patient discharge from hospital	1. Lower limbs active/passive activity<br>2. Lower limbs with elastic stockings<br>3. Pneumatic pump treatment	
	Early Off-bed activity and Ambulation	Rontine bed exercise and ambulation		Encourage early bed exercises and mobilization, with proper analgesia.	Bed exercises: 6 hours after awake<br>Early ambulation: 24 hours after surgery	√ Early mobilization
	Dressing change	POD 2: change dressing, observe the healing of incision, and remove the drainage tube if possible.		POD 2: change dressing, observe the healing of incision, and remove the drainage tube if possible.		
**Discharge**	Evaluation of surgical incision healing					
	evaluation of the quality of life	KPS score, satisfactory questionnaire		KPS score, satisfactory questionnaire		
	Evaluation of nutritional status when discharging	Nutritional assessment		Nutritional assessment	NRS2002, nutritional status assessment, PG-SGA	
	Mental state assessment	Anxiety and depression evaluation	Hospital Anxiety and Depression Scale (HADS)	Anxiety and depression evaluation	Hospital Anxiety and Depression Scale (HADS)	

Supplementary file 3. ERAS Protocol For Elective Craniotomies

Phase	Items	Control group		ERAS group		ERAS recommendation elements
		Content	Procedures	Content	Procedures	
**Follow up**	evaluation of the quality of life	2 weeks & 4 month after discharging from hospital	Out-patient revisit	2 weeks & 4 month after discharging from hospital	Out-patient revisit	
**Audit**	Audit	Assessing impact and encouraging compliance		Assessing impact and encouraging compliance		√ Audit

ERAS: Enhanced recovery after surgery

KPS: Karnofsky Performance Status Scale (KPS)

VAS: Visual Analogue Scale

VTE: venous thromboembolism

DVT: Deep vein thrombosis

NRS 2002: Nutritional risk screening 2002

PG-SGA: Patient-Generated Subjective Global Assessment

PONV: Postoperative nausea and vomiting

POD: postoperative day

NSAIDS: Non-Steroidal Anti-inflammatory Drugs

PPI: proton pump inhibitors, PPIs

AED: antiepileptic drug.
